# Cytotoxicity of 3D‐printed, milled, and conventional oral splint resins to L929 cells and human gingival fibroblasts

**DOI:** 10.1002/cre2.592

**Published:** 2022-05-15

**Authors:** Ralf Bürgers, Andrea Schubert, Jonas Müller, Sebastian Krohn, Matthias Rödiger, Andreas Leha, Torsten Wassmann

**Affiliations:** ^1^ Department of Prosthodontics University Medical Center Göttingen Göttingen Germany; ^2^ Department of Medical Statistics University Medical Center Göttingen Göttingen Germany

**Keywords:** cytotoxicity, 3D printing, milling, splint resins

## Abstract

**Objectives:**

Evidence on the biocompatibility of three‐dimensional (3D)‐printed and milled resins for oral splints is limited. This in vitro study assessed the influence of the manufacturing method on the cytotoxicity of oral splint resins on L929 cells and human gingival fibroblasts (GF1).

**Materials and Methods:**

Standardized specimens of four 3D‐printed, two‐milled, one‐thermoformed, and one‐pressed splint resin were incubated with L929 and GF1 cells for 24 h. Immunofluorescence and WST‐8 assay were performed to evaluate cytotoxic effects. One‐way analysis of variance and Tukey's multiple comparison test were applied with the variables “splint resin” and “manufacturing method” (*p* < .05).

**Results:**

Immunofluorescence showed attachment of L929 and GF1 cells to the splint resins. Irrespective of the manufacturing method, the WST‐8 assay revealed significant differences between splint resins for the viability of L929 and GF1 cells. L929 cells generally showed lower viability rates than GF1 cells. The evaluation of cell viability by the manufacturing method showed no significant differences between 3D printing, milling, and conventional methods.

**Conclusions:**

The cytotoxic effects of 3D‐printed, milled, and conventional oral splint resins were similar, indicating minor influence of the manufacturing method on biocompatibility. Cytotoxicity of the resins was below a critical threshold in GF1 cells. The chemical composition might be more crucial than the manufacturing method for the biocompatibility of splint resins.

## 1 INTRODUCTION

Oral splints are widely used in dentistry for the treatment of temporomandibular disorders (Ebrahim et al., 2012; Klasser & Greene, 2009), to protect teeth from clenching or grinding (Melo et al., 2019), or for orthodontic alignment therapy (Borda et al., 2020; Weir, 2017). While acrylic resins remain the “gold standard” material for oral splints, conventional splint manufacturing methods have been increasingly replaced by computer‐aided designing and computer‐aided manufacturing (CAD/CAM) methods over the past decade (Alghazzawi, 2016; Dedem & Türp, 2016). Traditionally, oral splints have been manufactured by vacuum thermoforming or by pressing cold‐ or heat‐cured acrylic resins on plaster casts of dental arches. Digital workflows, instead, are performed by intraoral scanning of dental arches, software‐supported splint design, and computer‐aided splint manufacturing (Dedem & Türp, 2016; Salmi et al., 2013; Vandenberghe, 2020). The two common CAD/CAM techniques used to manufacture oral splints are milling and three‐dimensional (3D) printing. Milling is a subtractive process carried out with the aid of CNC (computerized numerical control) equipment, in which oral splints are carved from prefabricated resin blanks produced under high pressure (Marcel et al., 2020). 3D printing is an additive process in which liquid resin monomers are successively cured by visible light creating solid objects layer by layer (Dawood et al., 2015). CAD/CAM manufacturing is characterized by high accuracy, standardization, and reproducibility, while being time‐ and cost‐efficient (Beuer et al., 2008; Dedem & Türp, 2016; Marcel et al., 2020). Also, the mechanical properties of 3D‐printed and milled oral splints are suitable for clinical use (Berli et al., 2020; Huettig et al., 2017).

Oral splints are worn repeatedly and for several hours at a time, during which they interact with teeth, saliva, and the oral mucosa. Aside from mechanical and practical characteristics, biological aspects must be considered to fully evaluate their clinical performance. Biocompatibility is the ability of a material to perform its desired medical function without eliciting an undesirable systemic or local host response (Williams, 2008). The biocompatibility of acrylic resins has been discussed controversially in dental research (Ata & Yavuzyilmaz, 2009; Gautam et al., 2012; Goiato et al., 2015; Schweikl et al., 2006), and adverse host responses like local irritation and ulceration (Weaver & Goebel, 1980) and mucosal edema (Ruiz‐Genao et al., 2003), as well as burning mouth sensations (Ali et al., 1986), have been described in patients as a result of acrylic resin exposure.

Acrylic resins result from a polymerization process, in which monomers are converted into stable polymers via an addition reaction that is activated by heat, light, or chemical activators (Bayraktar et al., 2006; Braden, 1988). The conversion from monomers to polymers is never complete, inevitably resulting in residual monomers and other toxic chemical products such as methyl methacrylate, formaldehyde, methacrylic acid, benzoic acid, or dibutyl phthalate within the material (Gautam et al., 2012; Goiato et al., 2015; Singh et al., 2013; Wedekind et al., 2021). They leach into the surrounding saliva via diffusion and subsequently interact with the host mucosa where they may have cytotoxic effects (Kurt et al., 2018; Singh et al., 2013; Urban et al., 2009).

The extent of residual monomers is influenced by the polymerization method underlying the manufacturing process of the resin material (Bayraktar et al., 2006; Wedekind et al., 2021). Dental materials manufactured by milling from highly polymerized resin blanks show levels of residual monomers comparable to those of conventional materials (Steinmassl et al., 2017). In contrast, 3D‐printed dental devices cured by light in a stepwise process were found to contain higher levels of residual monomers (Alifui‐Segbaya et al., 2019; Wedekind et al., 2021). Therefore, it appears possible that 3D‐printed oral splint materials exert higher cytotoxicity than milled ones, but, surprisingly, scientific evidence is scarce.

The aim of the present in vitro study was to investigate the cytotoxicity of commercially available 3D‐printed, milled, and conventional resin‐based oral splint materials with particular consideration of the manufacturing method. Besides ISO‐10993‐5 conform L929 mice cells, human gingival fibroblasts (GF1) were investigated to increase the transferability of the results to human oral tissue.

## 2 MATERIALS AND METHODS

### 2.1 Specimen preparation

Cylindrical specimens with a diameter of 10 mm and a height of 2.5 mm were manufactured from four 3D‐printed, two‐milled and two conventional (one thermoformed, one pressed of cold‐cure resin) splint resins (Table 1). As previously described (Schubert et al., 2021), rods of each resin were produced according to the manufacturer's instructions and sliced into disks using a separating machine (Micracut 201; Metkon, Bursa, Turkey). Surface polishing of the specimens was performed with an automated grinding machine (Digiprep 251, Metkon) and silicon carbide grinding paper.

**Table 1:** Specification of the oral splint resins assessed in this study

Manufacturing method	Product	Manufacturer
3D printing	Med610	Stratasys, Eden Prairie, MN, USA
	V‐Print splint	Voco, Cuxhaven, Germany
	FREEPRINT ortho 385	Detax, Ettlingen, Germany
	Dental LT Clear	Formlabs, Somerville, MA, USA
Milling	M‐PM crystal	Merz Dental, Luetjenburg, Germany
	Therapon Transpa	Zirkonzahn, Gais, Italy
Thermoforming	Erkodur	Erkodent, Pfalzgrafenweiler, Germany
Pressing	PalaXpress ultra	Kulzer, Hanau, Germany

### 2.2 Cell culture

L929 cells (lot. no. 85011425; Sigma‐Aldrich, St. Louis, MO, USA) complying with the ISO 10993‐5 standard for cytotoxicity testing were cultured in Dulbecco's modified Eagle's medium (Thermo Fisher Scientific, Waltham, MA, USA) with 10% fetal calf serum (Invitrogen, Darmstadt, Germany) and 1% penicillin–streptomycin (Fisher Scientific, Schwerte, Germany) under standard conditions. Immortalized human gingival fibroblasts (GF1) were established by the group for Oral Biology and Tissue Regeneration in our Department (ethic vote no. 16/6/2009) and have been published previously (Schubert et al., 2019).

### 2.3 Absorption assay

Cell viability of L929 and GF1 cells was assessed using WST‐8‐based assays (Cell Counting Kit 8 [CCK‐8]; Dojindo Molecular Technologies, Kumamoto, Japan). Under sterile conditions, specimens of the test resins (*n* = 25) were fixed to well bottoms of 24‐well plates using silicone. A total of 4 × 10^4^ L929 or GF1 cells (passages 3–8), respectively, in 1 ml culture medium were added to each well. Adherence was verified via light microscopy. After 24 h of incubation, the medium was removed from the wells and replaced by 600 µl of CCK‐8 detection solution at a dilution of 1:10 in the culture medium. After 4 h of incubation, the supernatant was transferred to another 24‐well plate without test specimens. Absorption was measured using a plate reader (Fluostar Optima; BMG Labtech, Ortenberg, Germany) at 450 nm and a reference wavelength of 650 nm. Standard glass specimens served as controls.

### 2.4 Immunofluorescence

Exemplary fluorescence imaging of L929 and GF1 cells on the surface of the resins was performed via immunocytochemistry. Resin specimens were incubated with 2 × 10^4^ of the respective cells for 24 h. After washing twice with phosphate‐buffered saline (PBS Tablets, Calbiochem, Merck, Darmstadt, Germany), cells were fixated using 2% paraformaldehyde solution for 15 min, treated with 0.25% Triton X‐100 (T8787; Sigma‐Aldrich) in PBS for 10 min, and blocked in 1% bovine serum albumin (BSA; Sigma‐Aldrich) in PBS for 15 min. A mouse monoclonal anti‐vinculin antibody (V9131; Sigma‐Aldrich) at a dilution of 1:1000 in 1% BSA in PBS was applied for 60 min at 37°C, followed by a polyclonal goat anti‐mouse secondary antibody to IgG (AlexaFluor 555, ab150114; Abcam, Cambridge, UK) and 4′,6‐diamidino‐2‐phenylindole (DAPI, 71‐03‐00; Kirkegaard & Perry Laboratories, Gaithersburg, MD, USA) at a dilution of 1:1000 in 1% BSA in PBS for 30 min at 37°C. Alexa Fluor 488 Phalloidin (A12379; Thermo Fisher Scientific) was incubated at a dilution of 1:400 in 1% BSA in PBS for 30 min at room temperature. Thorough washing with PBS was performed between all of the above steps. Specimens were dried and mounted on object slides for visualization via fluorescence microscopy (BZ‐X710; Keyence, Osaka, Japan). To reduce the autofluorescence of the test materials, haze reduction was applied to the images.

### 2.5 Statistical analysis

For the analysis of cell viability, values were tested for normal distribution using Q–Q plots. One‐way ANOVA was performed for the variables “splint product” and “manufacturing method.” Post hoc testing was performed via Tukey's multiple comparison test.

Statistical analyses were executed using GraphPad Prism 9 (GraphPad Software, San Diego, CA, USA). The overall level of significance was set at *α* = .05. Data from the WST‐8 assay are shown as medians with box–whisker plots.

## 3 RESULTS

**Figure 1:**
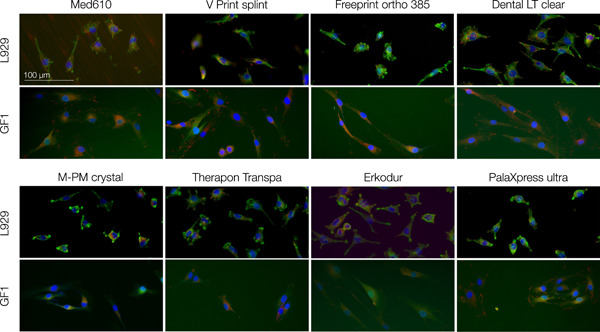
Fluorescence imaging of L929 and GF1 cells on the surface of dental splint resins after 24 h of incubation. Exemplary visualization shows adhesion of the cells to all of the tested resins. Morphology of GF1 cells includes more distinctive cell protrusions than L929 cells, which are generally rounder in shape. DAPI (blue) indicates cell nuclei, phalloidin (green), and vinculin (red) are associated with actin filaments of the cytoskeleton. DAPI, 4′,6‐diamidino‐2‐phenylindole.

Fluorescence imaging revealed the attachment of L929 and GF1 cells to all of the investigated splint resins after 24 h of incubation (Figure 1). Visualization of the actin filaments of the cytoskeleton via vinculin and phalloidin showed typical morphological characteristics of vital fibroblasts in monolayer culture. L929 cells were mostly round in shape, while GF1 cell bodies had a stretched out morphology with cell protrusions. Cell nuclei were visualized via DAPI.

**Figure 2:**
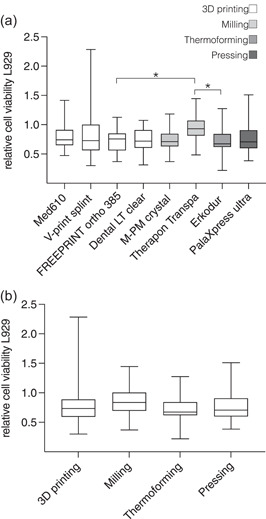
Relative cell viability of L929 cells according to the Cell Counting Kit ‐8 assay after 24 h of incubation with the investigated oral splint resins. (a) Cell viability shows significant differences between resins indicated by asterisks (*p* < .05). (b) Arrangement of the data according to the manufacturing method shows no significant differences. Glass was used for normalization (=1.0).

Cell viability was quantified according to the relative absorption of a WST‐8‐based assay. For L929 cells (Figure 2a), the highest cell viability was found for Therapon Transpa (milling, arithmetic mean 0.960, standard deviation ± 0.236), and it was significantly higher (*p* < .05) than for FREEPRINT ortho 385 (3D printing, 0.724 ± 0.207) and Erkodur (thermoforming, 0.705 ± 0.216). Overall, Erkodur was associated with the lowest L929 viability. There were no significant differences within the 3D‐printed or milled resins. To investigate the influence of the manufacturing method on cell viability, splint resins were grouped according to the manufacturing method, and there were no significant differences between 3D printing, milling, thermoforming, and pressing (Figure 2b).

**Figure 3:**
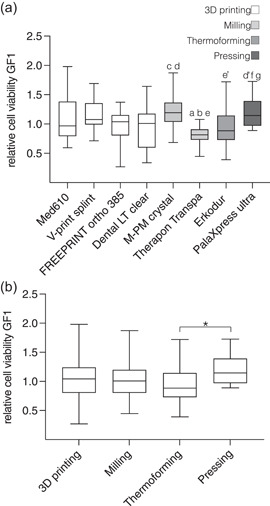
Relative cell viability of GF1 cells according to the CCK‐8 assay after 24 h of incubation with the investigated oral splint resins. (a) Cell viability shows significant differences between resins. ^a^ *p* < .05 compared with Med610, ^b^ *p* < .01 compared with V‐print splint, ^c^ *p* < .05 compared with FREEPRINT ortho 385, ^d^ *p* < .01 compared with Dental LT clear, ^d^^′^ *p* < .05 compared with Dental LT clear, ^e^ *p* < .0001 compared with M‐PM crystal, ^e^^′^ *p* < .01 compared with M‐PM crystal, ^f^ *p* < .001 compared with Therapon Transpa, and ^g^ *p* < .05 compared with Erkodur. (b) Arrangement of the data according to the manufacturing method shows significant differences (*p < .05) between thermoforming and pressing. Glass was used for normalization (=1.0).

For GF1 cells (Figure 3a), the highest (M‐PM crystal, 1.236 ± 0.312) and the lowest (Therapon Transpa, 0.813 ± 0.160) relative cell viability were found within the group of milled resins, and their difference was highly significant (*p* < .0001). No significant differences were revealed within the 3D‐printed resins. Erkodur (thermoforming, 0.922 ± 0.323) was associated with significantly lower GF1 cell viability than M‐PM crystal (milling) (*p* < .01). PalaXpress ultra (pressing, 1.201 ± 0.261) was related to significantly higher cell viability than Dental LT clear (3D printing, 0.921 ± 0.359, *p* < .01), Therapon Transpa (milling, *p* < .001), and Erkodur (thermoforming, *p* < .01). Arrangement of the results according to the manufacturing method indicated significantly higher (*p* < .05) cell viability of GF1 cells for pressing than for thermoforming (Figure 3b). 3D printing and milling showed no significant differences compared with conventional methods.

## 4 DISCUSSION

In the present study, the cytotoxic effect of 3D‐printed, milled, thermoformed, and pressed oral splint resins on L929 and GF1 cells was investigated. It has been suggested that 3D‐printed resins may be less biocompatible than milled or conventional resins due to higher amounts of residual monomers (Alifui‐Segbaya et al., 2019; Wedekind et al., 2021), but there is little evidence to support this. Therefore, the influence of the manufacturing method on biocompatibility of splint resins was evaluated for the first time.

In accordance with the current ISO 10993‐5 standard for cytotoxicity testing of medical devices, murine L929 cells were investigated in a monolayer cell culture model (Eljezi et al., 2017; Schmalz & Galler, 2017; Schubert et al., 2019; Tsuchiya et al., 1994; Wataha, 2012). In vivo, oral splints interact with the oral mucosa, which is composed of keratinocytes and underlying fibroblasts. Therefore, to simulate more tissue‐like conditions, we additionally assessed GF1 cells, a cell line of human gingival fibroblasts previously used to evaluate cytotoxic effects of composite resins (Schubert et al., 2019).

Fluorescence staining showed the attachment of L929 and GF1 cells to all of the investigated splint resins after 24 h. The actin cytoskeleton was visualized by labelling with phalloidin, a fluorescent toxin interacting with F‐actin filaments without affecting cell viability (Adtani et al., 2019; Barak et al., 1981; Chazotte, 2010; Wulf et al., 1979), and an antibody against vinculin, a small protein binding F‐actin in focal adhesions (Golji & Mofrad, 2013; Thievessen et al., 2013; Wilkins & Lin, 1982). Both cell lines showed the typical spindle morphology of fibroblasts in monolayer culture with L929 cells being smaller and slightly rounder in shape. Cell nuclei of L929 and GF1 cells were indicated by DAPI staining and showed full integrity. These findings suggest that none of the investigated resins exerted cytotoxic effects to an extent that inhibited cell attachment or affected morphology.

For the quantification of cytotoxic effects, CCK‐8 assay was performed. It contains WST‐8, a water‐soluble tetrazolium salt, which, in viable cells, is reduced to a colored formazan dye by dehydrogenase activity (Ishiyama et al., 1997). It represents a well‐established and reproducible method for the quantification of cell viability and cell proliferation (Camassa et al., 2021; Schubert et al., 2019; Zhang et al., 2019; Zhu et al., 2019), and it is more sensitive than other colorimetric assays (Failli et al., 2013; Miyamoto et al., 2002).

The cytotoxicity of resin products is influenced by the amount of leachable substances, especially residual monomers, that interact with the surrounding tissues. Residual monomers result from incomplete polymerization, and the degree of polymerization is affected by the method used to manufacture the resin (Bayraktar et al., 2006; Bural et al., 2011; Gautam et al., 2012; Schmalz & Galler, 2017). Layer‐by‐layer polymerization of resins by 3D printing has been associated with high rates of residual monomers, while milling from highly prepolymerized resin blanks has been related to low monomer levels (Alifui‐Segbaya et al., 2019; Steinmassl et al., 2017). Hence, it seemed possible that residual monomers leached from the 3D‐printed resins into the surrounding cell culture medium and affected biocompatibility in the present study. However, the biocompatibility of 3D‐printed and milled resins was similar and comparable to conventional resins. It is noteworthy that within the 3D printing group, the printing technology also had no decisive effect on biocompatibility: several technologies with importance to clinical dentistry were included in the present investigation, namely, poly jet modelling (Med610), digital light processing (V‐Print Splint, FREEPRINT ortho 385) and stereolithography (Dental LT clear) (Alghazzawi, 2016; Dawood et al., 2015), but showed no significant differences in cytotoxicity.

GF1 cell viability rates (arithmetic means) for all splint resins were greater than 75% compared to the control, and, therefore, their cytotoxic effect can be considered minor. Bural et al. (2011) L929 cells, however, showed a viability of greater than 50%, but less than 75% for FREEPRINT ortho 385 (3D printing), Dental LT clear (3D printing), M‐PM crystal (milling), and Erkodur (thermoforming), indicating slight cytotoxicity of these resins according to ISO 10993‐5.

Wedekind et al. (2021) showed that residual monomers and additives eluted from 3D‐printed, milled, and conventional splint resins had a substantial cytotoxic effect on human gingival fibroblasts. However, these data were obtained for a worst‐case scenario in terms of splint size, so comparability with our findings is limited.

Due to a lack of further evidence on oral splint resins, we related our results to studies on the biocompatibility of denture base resins as they are chemically similar. Srinivasan et al. (2018) showed that milled resins were equally biocompatible as conventional heat‐cured resins in an in vitro setting with human primary osteoblasts and mouse embryonic fibroblasts. In another study, cytotoxic effects of 3D‐printed denture base resins on L929 cells were negligible (Tzeng et al., 2021). In line with these findings, our data indicate that milling and 3D printing did not have a significant influence on the biocompatibility of splint resins. Instead, it can be assumed that the individual chemical composition of the studied splint resins was more relevant for cytotoxicity than the manufacturing method. This was evident in the milling group, where Therapon Transpa was significantly less cytotoxic to GF1 cells than M‐PM crystal. Dental resins differ by the type of monomers, the initiators used to start the polymerization reaction, and the additives incorporated to modify the mechanical properties, all of which may affect biocompatibility (De Matteis et al., 2019; Goiato et al., 2015; Jiao et al., 2015; Lee et al., 2020). Elution of unreacted chemicals was outside the scope of this study, but future investigations may help to understand the differences in resin toxicity that have been demonstrated.

L929 and GF1 cells responded differently to exposure to oral splint resins, with GF1 cells tending to exhibit higher relative cell viability. Several previous studies have shown that cells from different mammalian species and even between different donors within one species have varying sensitivities to eluted substances from dental resins (Engelmann et al., 2002; Schubert et al., 2019; Susila & Balasubramanian, 2016). Standardization of biocompatibility testing is therefore useful to improve the comparability of investigations, but the significance of results obtained from evaluations in murine L929 cells is limited because of their poor transferability to human cells. In addition, established in vitro cytotoxicity assays performed in monolayer cell cultures do not fully reflect the complex biological reactions in the oral mucosa (Schmalz & Galler, 2017; Wataha, 2012). Due to these limitations of the present study, our data must be considered preliminary. The need for improved biocompatibility testing could be addressed by using tissue‐engineered human oral mucosa with fibroblasts and keratinocytes in multilayer cell culture models that simulate organotypic conditions (Buskermolen et al., 2016; Izumi et al., 2000; Kinikoglu et al., 2009; Masuda, 1996; Moharamzadeh et al., 2008). In the long term, in vivo investigations will be required for final data validation.

Within the limitations of the present in vitro investigation, we conclude that the cytotoxic effects of 3D‐printed, milled, and conventional oral splint resins are comparable indicating minor effects of the manufacturing method on biocompatibility. In human gingival fibroblasts, cytotoxicity of all resins was below a critical threshold. Nonetheless, splint resins should be selected carefully for clinical application as individual biocompatibility may vary significantly, possibly due to different chemical compositions. Further in vitro and in vivo investigations are needed to validate our findings.

## AUTHOR CONTRIBUTIONS

**Ralf Bürgers**: Conceptualization; resources; writing—original draft; project administration. **Andrea Schubert**: Conceptualization; methodology; writing—original draft; visualization. **Jonas Müller**: Formal analysis; investigation. **Sebastian Krohn**: Visualization; writing—review and editing. **Matthias Rödiger**: Validation; writing—review and editing. **Andreas Leha**: Validation; formal analysis. **Torsten Wassmann**: Conceptualization; methodology; resources; writing—review and editing; supervision; project administration.

## CONFLICT OF INTEREST

The authors declare no conflict of interest.

## Data Availability

Data are available on reasonable request from the authors.
